# Studies analysing the need for health-related information in Germany - a systematic review

**DOI:** 10.1186/s12913-015-1076-9

**Published:** 2015-09-23

**Authors:** Dawid Pieper, Fabian Jülich, Sunya-Lee Antoine, Christina Bächle, Nadja Chernyak, Jutta Genz, Michaela Eikermann, Andrea Icks

**Affiliations:** Institute for Research in Operative Medicine, Witten/Herdecke University, Ostmerheimer Str. 200, Building 38, D- 51109 Cologne, Germany; Institute for Biometrics and Epidemiology, German Diabetes Center at the Heinrich-Heine University, Leibniz-Center for Diabetes Research, Düsseldorf, Germany; Public Health Unit, Faculty of Medicine, Heinrich-Heine University Düsseldorf, Moorenstraße 5, D-40225 Düsseldorf, Germany; Auf‘m Hennekamp 65, D-40225 Düsseldorf, Germany

**Keywords:** Information needs, Germany, Systematic review

## Abstract

**Background:**

Exploring health-related information needs is necessary to better tailor information. However, there is a lack of systematic knowledge on how and in which groups information needs has been assessed, and which information needs have been identified. We aimed to assess the methodology of studies used to assess information needs, as well as the topics and extent of health-related information needs and associated factors in Germany.

**Methods:**

A systematic search was performed in Medline, Embase, Psycinfo, and all databases of the Cochrane Library. All studies investigating health-related information needs in patients, relatives, and the general population in Germany that were published between 2000 and 2012 in German or English were included. Descriptive content analysis was based on predefined categories.

**Results:**

We identified 19 studies. Most studies addressed cancer or rheumatic disease. Methods used were highly heterogeneous. Apart from common topics such as treatment, diagnosis, prevention and health promotion, etiology and prognosis, high interest ratings were also found in more specific topics such as complementary and alternative medicine or nutrition. Information needs were notable in all surveyed patient groups, relatives, and samples of the general population. Younger age, shorter duration of illness, poorer health status and higher anxiety and depression scores appeared to be associated with higher information needs.

**Conclusion:**

Knowledge about information needs is still scarce. Assuming the importance of comprehensive information to enable people to participate in health-related decisions, further systematic research is required.

**Electronic supplementary material:**

The online version of this article (doi:10.1186/s12913-015-1076-9) contains supplementary material, which is available to authorized users.

## Background

Providing health-related information to the public and, more specifically, to patients and their relatives, can empower them to make informed decisions concerning prevention, screening and treatment [1]. Public involvement in healthcare decisions is regarded to be an essential element of high quality care [2, 3]. Related to this, the concept of empowering patients to participate as active partners in health-related decisions in terms of Shared Decision Making (SDM) has gained more prominence in the last years [4]. Therefore, a crucial prerequisite is the provision of information to the patient meeting his demands. Moreover, not only information targeting ill patients is needed, also ‘healthy people’ need health-related information, e.g. to decide whether they should participate in prevention or screening interventions.

Hence, exploring information needs is necessary to better tailor information to the specific needs of the target population and should thus be regarded as a prerequisite to the development of patient information.

Several systematic reviews exist investigating information needs [4–6]. However, prior work mainly concentrated on a single condition, in particular cancer, and involved patients [5, 6] or partners and family members, however, did not assess information needs in the general population [4].

Therefore, we conducted a systematic review on information needs independent of certain diseases in patients and relatives as well as the general population in Germany. We were particularly interested in the following questions:

1. For which diseases information needs were investigated
2. Which methodologies were used?

And regarding the findings of the studies

3. What are the health-related topics of information needs that the general population or patients are interested in?
4. How is the extent of (unmet) health-related information needs expressed by the investigated populations or patients?
5. How are personal or disease-related characteristics (e.g. age, gender, course of disease, etc.) or other variables associated with the desire for more information or the need for specific information about single topics?

## Methods

Studies were identified by searching the bibliographic databases Medline (via Embase), Embase (via Embase), Psycinfo (via EBSCO) and all databases of the Cochrane Library. Information needs may change over time, for example due to open access to a wide range of information or changes in the health care system. To obtain current information needs, the search was limited to a publication date from January 2000 to August 2012. There was no protocol for this systematic review.

### Definitions

The definition of information needs is ambiguous. In this review we define information needs as the ‘*recognition that their knowledge is inadequate to satisfy a goal, within the context/situation that they find themselves at a specific point in the time*’ [7].

### Inclusion and exclusion criteria

A study was included in the review if:

1. information needs were investigated;
2. the study participants were at least 18 years old;
3. the focus was on patients, parents or other family members of patients or the general population;
4. the study population lived in Germany; and
5. the full-text publication was written in German or English.

Systematic reviews were excluded. Studies that focused on information seeking or information preferences instead of information needs were excluded.

### Study selection process

All titles and abstracts were screened independently by two authors. The full-texts of potentially eligible articles were obtained. The references of the included studies were checked for further potentially relevant publications. Two reviewers assessed the eligibility of the full-texts according to the review inclusion criteria. Any disagreements were resolved by discussion. To identify studies performed in Germany we developed a geographic search filter (and applied it to each database) based on a search filter for retrieving studies performed in Spain [8]. The sensitivity of the search filter was increased by using more field descriptors in addition to searching for country-specific geographic names in the affiliation. The full search strategies for the individual database providers can be found in Additional file 1: Appendix 1.

#### Data collection process

Information on study type and design, population and methods of data collection was extracted from all included studies in standardized summary tables by one reviewer and checked by a second reviewer. To explore our main research questions, the following information was systematically extracted and analysed using/applying a descriptive content analysis (one reviewer coded key study findings, while a second reviewer verified and discussed them for each article and research question) based on predefined categories:

1. Diseases for which information needs were investigated
2. Methodology which has been used, as: qualitative or quantitative study, cross sectional or longitudinal study, assessment by written questionnaire or interview, closed or open-ended questions.

And regarding the findings of the studies

3. health-related topics of information needs. The topics were grouped into 12 categories based on a scheme originally developed by Rutten et al. for cancer patients [6]. The scheme was adopted and applied irrespective of health condition (Table 1). Regarding the extraction and analysis we distinguished if topics of information or single items were predefined (e.g. rating or choice task, dichotomous questions) or if participants had to state or express their need for information by themselves (e.g. open ended questions). Data was extracted for this question, if at least two categories were analysed in a study.

**Table 1:** Categories of information needs

Category	Information needs about (examples)
Aetiology	Causes of disease, risk factors, individual risk
Complementary and alternative medicine	Complementary and alternative medicine
Coping	Coping with pain, handling of disease in daily life, psychological support, spiritual support, self-help group
Diagnosis	Diagnostic procedures, examination results, progress of disease, symptoms
Financial/legal	Financial support, reimbursement of health insurance, entitlement for disabled people, social law, applying for pension, sick leave certification
Medical system	Contact data of different health care providers, quality data about health care providers, services of health insurances, ‘How many patients with a specific condition does the doctor treat’
Nutrition	Diet
Prevention/Health promotion	Screening tests, protection against risk factors, What can I do by myself
Prognosis	Course of disease, possible consequences
Rehabilitation	Possibilities of rehabilitation, clinics, payer, contact persons
Social life/interpersonal	Impact of disease on job, school, free time, daily life, partnership, sexuality
Treatment	Current treatment, treatment options, risks, benefits, advantages and disadvantages, side effects, physiotherapy
4. whether and how the extent of information needs was evaluated, i.e. with regard to medical information in general or about specific topics (e.g. ratings of predefined topics or proportion of people who claim to have unmet information needs).
5. whether and how associations between information needs and other variables (e. g, anxiety, depression, disease activity, duration of illness, education) or patient characteristics (e.g., age, gender, course of disease, etc.) were explored.

We did not assess quality of the included studies due to a lack of an established and validated critical appraising tool for the expected study designs.

This study did not need ethical approval nor was individual patient consent needed.

## Results

**Fig. 1:**
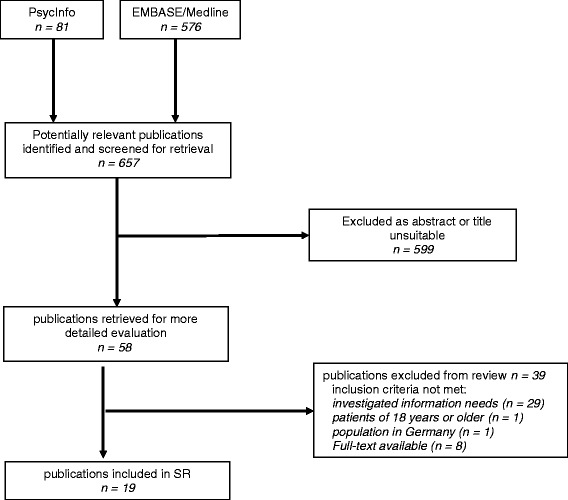
Flow chart of systematic search

The search strategy resulted in 657 hits, of which 19 studies [9–27] were included in our analysis (Fig. 1).

The characteristics of the studies are summarized in Table 2. Fourteen papers were published in the last 6 years which indicates a slightly increasing research interest.

**Table 2:** Overview of extracted studies

Author/year [Reference]	Aim	Design/Study type	Population	Methods/type of question	What was evaluated?	Predefined Topics	Results
Berth et al. (2007) [10]	To validate the German version of the Amsterdam Preoperative Anxiety and Information Scale (APAIS).	Cross-sectional design;	68 patients questioned before surgery on the lower extremities in the orthopedic department of a University Hospital.	Questionnaire for self-assessment of preoperative anxiety and the need-for information (APAIS).	Association between IN and:	Treatment	A higher level of need-for-information is accompanied by a higher level of anxiety (Spearman coefficient *r* = 0.59, *ρ* < 0.01). One exception is the very small group of male patients in which the anxiety level does not significantly differ between the three groups of need-for-information.
		Validation study.	30.9 % male;	Likert scales.	• anxiety		
			Mean age (range) was 55.6 (18–85) years.				
Conrad et al. (2012) [11]	To identify topics of IN for patients with Crohn’s disease or ulcerative colitis.	Cross-sectional design;	612 patients with Crohn’s disease and 444 patients with ulcerative colitis.	Postal survey with 19 predefined categories of IN;	Topics of interest.	Aetiology	Most frequently expressed IN (≥70 %):
		Quantitative analysis of predefined topics.	34.8 % male;	Dichotomous question for each category:	Association between IN and:	CAM	• Treatment options (82.7 %)
			Mean age (SD) was 42.4 (12.8) years;	“Yes, I want more information about the topic…”	• age	Financial/legal	• Cause of disease (79.5 %)
			Duration of illness ≥ 10 years was 54.8 %.		• anxiety	Medical system	• What can I do myself (79.2 %)
					• depression	Nutrition	• CAM (76.7 %)
					• disease activity	Prevention/ health promotion	Gender differences were rare;
					• duration of illness	Prognosis	IN were significantly higher with decreasing age, increasing education, shorter duration of illness, higher illness activity and higher depression and anxiety scores.
					• education	Rehabilitation	
					• gender	Social life	
						Treatment	
Eustachi et al. (2009) [12]	To assess the use of, knowledge about and demand for complementary and alternative medicine (CAM) in cancer patients	Cross-sectional design	156 outpatient cancer patients at the tumor treatment centre of a university hospital.	Questionnaire including a 5 point Likert scale for “degree of being informed” and “subjective importance” of CAM treatment.	Extent of IN: demand for consultation about CAM.	CAM	48 % definitely demanded CAM consultation irrespective of whether they already used CAM or not.
		Quantitative analysis of predefined topics.	62.2 % male;		Association between IN and:		44 % designated their degree of being informed about CAM as poor or very poor.
			Mean age (range) was 60.1 (18–81) years;		• age		24 % of the patients neither used CAM nor were interested in consultation, 24 % did not use CAM but now requested information on this field.
			Mean (SD, range) time since cancer diagnosis was 34.8 (42.6, 0–239) months;		• health status		59 % rated CAM as important or very important for themselves.
			Frequent tumors: gastrointestinal system (20.8 %), breast (17.5 %) and lymphatic organs (17.5 %); Metastases (37.2 %).		• knowledge		IN were significantly higher with decreasing age, lower degree of being informed and poorer health status.
Geraedts/Amhof (2008) [13]	To analyze gender differences in the demand for quality related information on health care providers.	Cross-sectional design.	Representative sample of German adults (*N* = 1523);	Health Survey using 5 point Likert scales assessing the demand for quality-related information on physicians and health care providers (10 items) and hospitals (33 items).	Extent of IN: demand for quality related information on health care providers.	Medical System	Respondents expressed a high demand for quality-related information on medical specialists (89 %), hospitals (82 %) and GP/dentist (80 %).
		Quantitative analysis of predefined topics.	49 % male,		Association between IN and:		Regarding hospitals information needs were highest for qualification of physicians (98 %), cleanness (97 %), qualification of nursing staff (96 %), newest and best available treatments (96 %) and friendliness of staff (96 %).
			Age range was 18–79 years.		• age		Among all socio demographic variables considered, gender exerted the strongest influence on the responses. Women in comparison to men expressed a higher demand for quality-related information on health care providers, chose health care providers differently to some extent, and rated a multiplicity of criteria used to make a quality-oriented choice of hospitals more important than men. Hardly any significant differences were found for the factor age.
					• gender		
Heesen et al. (2007) [14]	To analyze the prerequisites for patient participation in decision making in patients with multiple sclerosis (MS).	Cross-sectional design.	169 multiple sclerosis (MS) patients;	Survey, no details given.	Topics of interests.	No details given.	Main interests related to alleviation of symptoms and magnetic resonance imaging, followed by knowledge about relapses, steroids and complementary medicine (CAM).
		No details given.	No details given.		Association between IN and:		Interests were largely influenced by disease stage and course (relapse-remitting vs. primary-progressive) and knowledge.
					• course of disease		
					• knowledge		
Himmel et al. (2005) [15]	To analyze the information requests of patients visiting an internet expert forum on involuntary childlessness.	Cross-sectional design.	513 answers from participants;	Questionnaire comprising 22 items (free text) related to reasons for visiting the website and the expert forum, the use of the information, the satisfaction with the experts’ answers and actual treatment situation.	Topics of interests.	Not specified a priori.	Reasons for visiting the website:
		Qualitative study.	99.2 % female;	Open ended questions.			72.9 % General information about involuntary childlessness, conception, or an evaluation of drugs
			Age range was 18–43 years.				45.1 % Current treatment
							32.1 % Different treatment options
							25.5 % Causes of infertility
							22.0 % Diagnostic data
							7.7 % Other
Jungbauer et al. (2008) [16]	To investigate the need for professional assistance in carers of stroke patients and how this need changes in the course of rehabilitation.	Longitudinal design.	Ten highly burdened spouses of stroke patients;	Spouses of stroke patients were interviewed twice: at the beginning of in-patient rehabilitation period (T1) and one year later during the outpatient rehabilitation (T2).	Extent of IN: desire of disease-related information.	Not specified a priori.	At the beginning of in-patient rehabilitation period, carers requested mainly disease-related information (e.g. about stroke, its prognosis, treatment and rehabilitation possibilities). Active provision of disease-related information by medical staff was desired.
		Qualitative study.	40 % male;	Interviews were analyzed using Grounded Theory coding procedures.	Topics of interest.		The desire for further disease-related information was seldom mentioned one year later during the out-patient rehabilitation period, whereas the need for emotional assistance increased. The need for information was more pronounced in female participants, who also requested such information more actively.
			Mean age was 61 years.	Open ended questions.	Association between IN and:		
					• duration of disease		
					• gender		
Knelangen et al. (2010) [17]	To identify the potential need for evidence-based health information.	Cross-sectional design.	Endometriosis:	Two online surveys.	Topics of interest.	Aetiology	Most interesting topics for endometriosis (rated as very interesting) were consequences (79 %), causes (73 %) and CAM (70 %).
		Quantitative analysis of predefined topics.	754 participants (73 % concerned, 8 % relatives);	Different information categories were assessed using a 6-point Likert scale with response categories from 1 = very interesting to 6 = not interesting at all.		CAM	Regarding skin cancer screening the topics self-diagnosis (83 %), self-protection (65 %) and causes/risk factors (59 %) were rated as very interesting most frequently.
			8 % male;	In addition open-ended questions were used.		Coping	
			Age range was 16–76 years.			Diagnosis	
			Skin cancer screening and prevention:			Prevention/ health promotion	
			265 participants;			Prognosis	
			43 % male;			Social life	
			Age range was 16–79 years.			Treatment	
Maywald et al. (2005) [18]	To evaluate unmet drug information needs in patients.	Cross-sectional design.	3316 inquiries from a catchment area covering 500.000 inhabitants;	All inquiries within 36 months were analysed via a standardized answer sheet to determine the type of counselling demand.	Topics of interest.	Not specified a priori.	The questions were mainly related to adverse drug reactions and interactions (26.6 %) as well as to common information on efficacy of specific therapies (27.2 %). Questions about (contra-) indication, self-medication, application/dosage, financial and legal questions were less frequent (<10 % respectively).
		Quantitative analysis of inquiries to a drug information service.	33.8 % male;	Open ended questions.			
			64.5 % were over 60 years old.				
Nickel et al. (2010) [19]	To explore the information needs of people dependent on care and their informal caregivers.	Cross-sectional design.	89 participants: 38 (43 %) information seeking family members, 17 (19 %) patients, 2 (2 %) patient’s friend, 1 (1 %) legally appointed carer; situation was not specified for 31 persons (35 %).	Semi-structured questionnaire which was analysed by qualitative content analysis	Topics of interest.	Not specified a priori.	Four major topics of IN were identified:
		Qualitative study.					Information on health care system: 30 (27.8 %)
							Information on individual access options to health care system: 31 (28.7 %)
							Information on regional service provider: 17 (15.8 %)
							Context-specific and disease-specific questions: 30 (27.8 %)
Neumann et al. (2011) [20]	To identify and predict subgroups of IN among cancer patients.	Cross-sectional design.	326 cancer patients suffering from bronchial (*n* = 28), oesophagus (35), colorectal (18), breast (109), prostate (54) and skin cancer (68);	A cancer-specific instrument for the German health system was developed: Cancer Patients Information Needs (CaPIN) measure consisting of 23 dichotomous items (yes/no) regarding the question "Looking back on your hospital stay, would you have liked more information about...."	Extend of IN: proportion of people with unmet IN.	Coping	Highest information needs (% yes) for the categories: methods of health promotion (54.2 %), medical examination results (47.7 %), nutrition (45.2 %), diagnosis and progress of cancer (43.3 %) and other treatment options (41.2 %).
		Quantitative analysis of predefined topics.	52 % male;	Latent Class Analysis (LCA) was used to identify subgroups sharing similar information needs.	Topics of interest.	Diagnosis	Five subgroups were identified with LCA: No unmet IN (31.4 %), high level of psychosocial IN (27 %), high level of purely medical IN (16 %), high level of medical and psychosocial IN (13.6 %) and high level of psychosocial IN (12 %).
			Mean age (SD, range) was 58.7 (11.2, 19–76) years.		Association between IN and:	Financial/legal	Most significant predictors for class membership were "trust in nurses", "caring attention from nurses" and "physician empathy", indicating fewer unmet IN. A higher age and no requirement of psychological support were also statistically significant predictors indicating fewer unmet IN.
					• age	Nutrition	
					• caring attention from nurses	Prevention/ health promotion	
					• course of disease	Prognosis	
					• education	Social life	
					• gender	Treatment	
					• physician empathy		
					• requiring psychological support		
					• trust in nurses		
					• working status		
Oskay-Özcelik et al. (2007) [21]	To explore breast cancer patients' information needs with a special focus on doctor-patient communication.	Cross-sectional design.	*N* = 617 cancer patients (552 via online questionnaire, 65 via hard copy);	Online or hard copy questionnaire with 62 items in multiple choice format.	Topics of interest.	No details given.	Most frequent answers for information needs were:
		Quantitative analysis of predefined topics.	Median age (range) 48 (21–92) in the online group, 55 (40–92) in the hard copy group.	Question to explore information needs:			1) Am I getting the right therapy (89 %)?
			65 % with curative treatment.	'What do you think are the three most important items of information regarding your illness and its treatment?'			2) How many patients with my condition does my doctor treat (46 %)?
							3) Can I be enrolled into a trial (46 %)?
Richter et al. (2011) [22]	To analyze inquiries sent to an online ask-the-doctor service on a rheumatology website.	Cross-sectional design.	1133 inquiries of patients (60 %), relatives (24.3 %) and physicians (15.7 %);	Content analysis of web-based inquiries.	Topics of interest.	Not specified a priori.	Inquiries were most frequently related to the following topics: medication(indication, effects, side effects) (30.8 %), contact to a rheumatologist nearby (24.9 %), diagnosis-related questions (15.7 %), second opinion (11.6 %).
		Quantitative analysis of inquiries sent to an ask-the-doctor service.	37.8 % male;	Open ended questions.			Relatives addressed different topics and issues than patients.
			Mean age reported by 113 patients (SD, range) was 37.8 (12.6, 17–72) years.				
Steckelberg et al. (2004) [23]	To explore consumers' information needs and attitudes for informed choice on colorectal cancer screening.	Cross-sectional design.	50 participants, recruited by announcements in local newspapers;	Focus group discussion, questionnaire with semi-structured questions and open ended questions.	Extent of IN: rating of topics.	Not specified a priori.	The six most relevant topics: screening methods in general (1.5 ± 1.1) , therapy of colorectal cancer (1.8 ± 1.4), prevention of colorectal cancer (1.9 ± 1.5), nutrition (1.9 ± 1.4), symptoms of colorectal cancer (2.0 ± 1.1), anatomy and physiology (2.0 ± 1.0).
		Qualitative study to explore possible relevant topics of interest.	30 % male;	Relevance of identified topics were rated with a 6-point Likert scale (1 = high relevance, 6 = low relevance).	Topics of interest.	Diagnosis	Least relevant topic was sponsoring (2.9 ± 1.6).
		Quantitative analysis of identified and clustered topics.	Mean age (SD) was 59 (10.6) years;			Nutrition	
			34 participants have taken part in colorectal cancer screening before.	Relevance of identified topics were rated with a 6-point Likert scale (1 = high relevance, 6 = low relevance).		Prevention	
						Treatment	
Thon/Ullrich (2009) [24]	To assess sources of information and information needs in parents of children with a rheumatic disease.	Cross-sectional design	116 families continuously attending a paediatic rheumatology outpatient clinic;	Questionnaire with a 4-point Likert scale for 15 pre-selected topics/items regarding	Extend of IN: interest in further information.	Aetiology	Overall, parents considered themselves well-informed. However, their interest in further information was high almost irrespective of the amount of prior information. Three main response patterns were identified:
		Quantitative analysis of predefined topics.	31 % male (children);	1) the amount of prior information and	Topics of interest.	CAM	1) topics covered by prior information which were nonetheless of high interest: aetiology, prognosis, treatment and adverse effects;
			Mean age (SD) of children was 6.9 (4.3) years;	2) the amount of interest in further information.	Association between IN and:	Prognosis	2) topics with low prior information and of high current interest: complementary and alternative medicines (CAM), psychological impact, inpatient rehabilitation facilities, educational/vocational rehabilitation;
			Mean duration of disease was 2.6 (4.3) years.	Items were summed up in a information score and a interest score.	• knowledge (prior information)	Rehabilitation	3) topics with low prior information, but only moderate to low interest: entitlements for disabled people, implications on partnership and sexuality.
						Social life	
						Treatment	
Ullrich et al. (2003) [25]	To explore the information needs of parents of children with juvenile idiopathic arthritis (JIA).	Cross-sectional design.	118 parents of 121 children with JIA attending a paediatric rheumatology outpatient clinic;	Questionnaire with a 10-point Likert scale for the importance of detailed information about the JIA in general and the satisfaction of information provision.	Extent of IN: proportion of people with unmet IN.	Aetiology	All parents considered detailed information as very important (mean = 9,52; max = 10). The majority felt being well-informed, although 80 % mentioned at least one issue of further IN.
		Quantitative analysis of predefined topics and extent of unmet IN.	39 % male (children);	Parents could suggest a topic of interest in a free text. Additionally, they were asked to select (dichotomous question) predefined topics to which they would like more information.	Topics of interest.	CAM	Pre-defined topics with highest interest were aetiology (76,7 %), nutrition (72,2 %), side effects of drugs (70,1 %) and alternative medicines (69,8 %). Topics with lowest interest were self help (26,1 %) and psychological consultation (24,8 %). Parents were more satisfied with their physician and felt better informed had significantly fewer unmet IN.
			Mean age (SD) of children 10.3 (4.5) years;		Association between IN and:	Coping	In the free text section the topics prognosis and course of disease were mentioned most frequently. Topics differed dependent on the age of the children.
			Mean duration (SD) of disease 4.3 (3.2) years.		• age of children	Diagnosis	
			24 % male (parents)		• knowledge (degree of being informed)	Nutrition	
			Mean age (SD) of parents was 39.2 (6.8) years.		• satisfaction with physician	Prognosis	
						Rehabilitation	
						Social life	
						Treatment	
Vogel et al. (2008) [26]	To assess patients' information needs and experiences in the course of breast cancer treatment.	Longitudinal design.	135 women with first breast cancer diagnosis and no evidence of metastases;	Questionnaire with 8 items rated on a 5-point scale (5 = high IN; 1 = low IN) to assess information needs at the beginning of initial treatment with two follow-ups at 3 and 6 months	Extent of IN: rating of topics.	Diagnosis	Information needs were highest for treatment (4.1), and diagnosis (4.0) at baseline and highest for aftercare (4.0) and treatment (3.8) at 6 months follow-up.
		Quantitative analysis of predefined topics.	Mean age (SD, range) was 53,9 (10.9, 19–75) years.		Topics of interest.	Prognosis	Information needs for all topics decreased over time, except aftercare.
						Social life	Information needs for examination and medical tests did not change significantly over time.
						Treatment	
Vogt/Schäfer (2011) [27]	To identify counseling topics relevant to young women about combined oral contraceptives (COC).	Cross-sectional design.	30 selected women from a representative research panel;	Online questionnaire including a list of 25 potential counselling items(risks, benefits and fears).	Extent of IN: rating of topics	Treatment	The mean rating of interest for all 25 potential counselling items was 5. Items with high interest ratings (mean and CI ≥4) which were also seen as mandatory items in counselling were cervical cancer risk, change in sexual desire, depressed mood, sub fertility after discontinuation, weight gain, benign breast disease, pelvic inflammatory disease, dysmenorrhoea and acne.
		Qualitative and quantitative analysis of predefined topics.	Median age (range) was 20 (18–24) years.	Interest in various topics was rated on a 7-point Likert scale (1 = no interest; 7 = high interest).	Association between IN and:		No trend was observed for interest ratings dependent on different educational levels. Women who had no experience with usage of COC tended to report higher interest levels than current or past users. The relationship between interest and knowledge ratings about risks and benefits of combined oral contraceptives showed no clear trends
					• education		
					• experience		
					• knowledge		
Wildner et al. (2002) [28]	To assess citizens' perspective of patients' perceived IN.	Cross-sectional design.	Representative sample of general population (*n* = 3008);	CATI with trained interviewers.	Extent of IN: proportion of people with unmet IN.	Not specified a priori.	Of the 3008 people interviewed 1043 (35 %) said they had some kind of IN, 1437 (48 %) had no IN, 73 (2 %) did not answer and 455 (15 %) were not sure.
		Qualitative analysis of relevant categories of IN.	38.7 % male;	Responses to open ended questions were categorized.	Topics of interest.		Top five categories (*n* = 1043): musculoskeletal diseases 18.1 %, prevention/health promotion 15.4 %, cardiovascular diseases 8.2 %, cancer 5.9 % and sickness funds 5.8 %.
			Age was ≥18 years.		Association between IN and:		Younger people and people who received no medical care had significantly higher needs for information on prevention and health promotion. Gender differences were mentioned but were not obvious.
					• age		
					• gender		
					• patient status		

Information needs of patients were assessed in 12 studies [9, 10, 12, 13, 15–20, 24, 27], followed by information needs in the general population (4 studies) [11, 21, 25, 26], of spouses/family members (4 studies) [14, 15, 18, 20], and parents (2 studies) [22, 23].

### Diseases for which information needs were analysed

The diseases the information needs referred to were very heterogeneous. Only information needs regarding cancer (5 studies) [10, 17, 19, 21, 24] and rheumatic diseases (3 studies) [20, 22, 23] were assessed more than once. Other diseases were: injuries of lower extremities [27], Crohn’s disease and ulcerative colitis [9], multiple sclerosis [12], and stroke [14].

### Methodology used to assess information needs

The study design was cross-sectional in 17 [9–13, 15–23, 25–27] and longitudinal in 2 studies [14, 24]. A quantitative approach was chosen in 11 studies [9–11, 15–17, 19, 20, 22–24], while a qualitative analysis was found in 4 studies [13, 14, 18, 26]. Two studies combined a quantitative and a qualitative approach [21, 25]. In one study, an instrument was validated [27]. Information on the study design was missing for one study [12].

Questionnaires were used most often (12 studies) [9–11, 13, 15, 18, 19, 21–25]. Interviews were conducted in two studies [14, 26], a specific instrument (the Amsterdam Preoperative Anxiety [27] and Information Scale and Cancer Patients Information Needs [17]) or an information service (e.g. ask-the-doctor service) were used twice [16, 20], respectively. To assess the information needs, questions with rating scales (e.g. ‘Please rate how relevant/important/interesting, etc. the following topics are to you’) were applied in nine studies [10, 11, 15, 21–25, 27]. Open ended questions with a free text option were found in eight papers (e.g. ‘Are there specific topics to which you like more information?’) [13–16, 19–21, 23, 26]. Multiple choice (e.g. ‘What are the three most important topics…?’) [19, 23] and dichotomous questions (e.g. ‘Do you want more information regarding the following topics: (yes/no)’) were used twice [9, 17], respectively. One study did not provide sufficient details of methods or measures [12].

### Health-related topics of interest

In 15 studies an evaluation of information need topics was conducted [9, 12–24, 26]. Treatment was the most prominent topic of interest, including questions about the current treatment, treatment options, advantages and disadvantages of each alternative, possible side effects, etc. [12, 15–17, 19, 20, 22, 23, 25, 26]. However, it was also the most common predefined category (nine studies) [9, 15, 17, 21–25, 27]. Information on common topics such as etiology [12, 16, 18, 25], diagnosis [15, 16, 20, 23, 26], prevention/health promotion [12, 18, 20, 24] and prognosis [15, 17, 18, 25] was also frequently desired, irrespective of the kind of disease. They were considered as predefined topics in four [12, 15, 22, 23], five [15, 18, 21, 23, 24], four [9, 15, 18, 22] and six [9, 15, 18, 22–24] studies, respectively. Other, more specific topics such as complementary and alternative medicine (CAM) or nutrition were also of high interest, when considered as a predefined topic. Information on CAM was highly relevant for patients with inflammatory bowel diseases [9], cancer [10], multiple sclerosis [12], endometriosis [15], rheumatic diseases [22] and for parents of children with juvenile idiopathic arthritis (JIA) [25]. Participants of four studies reported an interest in information on nutrition [9, 18, 21, 24]. Information on coping, including psychological support and self-help groups was shown to be of moderate to high interest in three studies [15, 18, 23]. Information on the medical system (e.g. information on statutory health insurance, access to care, local care services) seems to be more important for women and people in need of care as well as their caregivers [9, 11, 18, 19]. Clinical trials were also a topic of interest in some studies [12, 19, 20]. In a survey regarding information about breast cancer and its treatment, 46 % of the participants chose the item ‘Can I be enrolled into a trial’ as one of the three most important items [19]. ‘Validity of studies’ was of particular interest in well informed patients with multiple sclerosis, while patients with lower knowledge scores did not mention the item among the first ten [12]. Neither of the latter studies provided any details about the considered predefined topics. For that reason the topic ‘clinical trials’ is not included in the catalogue of different categories (Table 1). The following topics rehabilitation [9, 22, 23], financial and legal issues [9, 16, 17] and impact on social life [9, 15, 17, 22–24] were also mentioned as a topic of interest.

### Extent of information needs and unmet needs

Ten studies contained data about the extent of information needs [10, 11, 14, 17, 21–26]. The need for more detailed medical information was reported frequently. The demand for information was substantial in the whole population as well as in specific patient groups and their relatives. In a survey, 35 % of the 3008 people from the general population reported that they had some kind of information need while almost half (48 %) declared no specific needs [26]. In another survey, Geraedts and Amhof showed that there was a high demand for information on the quality of health care providers in the general population [11]. Additionally, a total of 89 and 86 % of the surveyed sample wanted more information on the quality of medical specialists and hospitals, respectively. Analysing the information needs of young women regarding combined oral contraceptives the mean interest rating of all 25 potential counseling items was 5 on a 7-point-scale. All items had a rating of 4 or higher [25]. Similar results were observed in a study about consumers' information needs for informed choice on colorectal cancer screening, where participants rated all potential topics as relevant [21]. Identifying subgroups of information needs among cancer patients using latent class analysis (LCA) Neumann et al. found that 68.6 % of the patients had some kind of unmet information needs [17]. Vogel et al. explored information needs in breast cancer patients and found them to be time-dependent [24]. In a study on the demand for CAM among cancer patients, almost half (48 %) of the interviewed cancer patients demanded consultation about CAM irrespective of whether they already used CAM or not [10]. Only 24 % were not interested in CAM. In a qualitative survey, family members of stroke patients reported a need for detailed disease-specific information by the medical staff in a personal conversation during inpatient rehabilitation [14].

Among parents of children diagnosed with a disease, parents of children with rheumatic diseases had high interest in further information, although they considered themselves as being well informed [22]. Almost all parents (85 %) of children with JIA considered detailed information on the disease as very important. The mean value for importance was 9.52 on a 10-point-scale [23]. A total of 58 % of the parents had specific information needs for at least one theme. One fifth (22 %) did not indicate a specific theme and only 20 % stated that they did not have any specific information needs.

### Factors associated with information needs

Associations between information needs and other variables or characteristics were analysed in eleven studies (Table 2) [9–12, 14, 17, 22, 23, 25–27]. We found analyses concerning the association between information needs and gender, age, education, duration and course of the disease, knowledge, emotional factors (e.g. anxiety, depression) and the relationship between patient and health professionals.

In two studies, women expressed higher information needs than men [11, 14]. Assessing the need for quality-related information on health care providers, significant gender differences were found in 17 of 41 items [11]. In this study, gender was most strongly associated with the need for information among all considered sociodemographic variables. However, in other studies analysing information needs of cancer patients [17] and patients with inflammatory bowel diseases [9], hardly any gender differences were found.

There is some evidence that age influences type and extent of information needs. Conrad et al. [9], Eustachi et al. [10] and Neumann et al. [17] found higher information needs in younger patients. A trend for higher information needs in younger people was also observed in a survey of the general population [26]. In addition, younger people showed higher interest in disease prevention and health promotion [26]. Information needs of parents of children with JIA differed depending on the age of the child [23].

No conclusive association was found between information needs and education [9, 25], as hardly any significant differences were found between people with different levels of education.

Regarding the duration of disease, a negative correlation between information needs and the duration of an illness was found in two studies [9, 17]. The course of disease had a significant impact on the amount or type of information needs in several assessed studies. Neumann et al. stated that cancer patients in early disease stages had more unmet needs for information on results of medical examinations and treatment options, whereas patients in more progressive stages had more unmet needs for information on social issues and health promotion [17]. Vogel et al. have observed decreasing scores of information needs for all topics except ‘aftercare’ in the course of breast cancer treatment. Only one specific topic showed no significant change (*p* > 0.05) of interest in the course of treatment ('examination and medical test') [24].

Cancer patients with a poorer health status had higher information needs about CAM [10]. Heesen et al. detected considerable differences in information needs between primary-progressive (PP) and relapsing-remitting (RR) patients with multiple sclerosis. RR patients expressed a higher need for information regarding magnetic resonance imaging, relapses and CAM, while PP patients were more interested in the treatment of gait disturbances, physiotherapy and experimental therapies [12]. Patients with inflammatory bowel diseases had significantly higher information needs with increasing disease activity scores [9].

For the association between knowledge and information needs ambiguous results exist. According to Ullrich et al. parents of children with JIA had lower information needs when they had higher knowledge scores and were more satisfied with the information provision [23]. Likewise, cancer patients who felt informed had significantly lower information needs about CAM [10]. In a small sample of young women, however, the relationship between interest and knowledge ratings about risks and benefits of combined oral contraceptives showed no clear trends [24]. Assessing the information needs in parents of children with a rheumatic disease, the level of prior information (self-rating) and the interest in further information were compared [22]. All topics which were covered by prior information were nonetheless of high interest. No response pattern for topics covered by prior information and low current interest was detected. Patients with multiple sclerosis and knowledge scores in the upper quartile rated the item ‘validity of studies’ among the first three of interest while the remaining patients did not mention this item among the first ten [12].

Anxiety, depression and the requirement for psychological support were correlated with increased needs for information in three studies [9, 17, 27].

Patients had less unmet need for information when they were satisfied with their actual treating physicians [23], trusted their nurses highly, received more caring attention from nurses or were treated empathically by their physicians [17].

## Discussion

This systematic review investigated the methodologies used to assess information needs, and identified findings from existing studies regarding topics of health-related information needs, the extent of health-related information needs, and associations between disease-related characteristics or other variables and the desire for more information or the need for specific information about single topics.

### Methodological aspects of the assessment of information needs in the identified publications

Studies on information needs are rare for many diseases (except for cancer) but a recent increase in publications indicates rising interest.

In the identified articles a wide range of methods was described: qualitative research employing focus groups [21], quantitative methods using questionnaires and Latent Class Analysis (LCA). The most frequently used measure to assess the need for information was the Likert scale [10, 11, 15, 21–23, 25, 27]. Some methodological issues regarding the usage of Likert scales in this context have been discussed [28, 29]. Ratings for every single item may lead to biased results because participants tend to rate every item as important [30]. Hence, mean values of single information categories do not differ much within single studies and tend to reach high levels. Such ceiling and cluster effects can also be found in several of the assessed studies in this review [14, 18, 24, 26] and make it challenging to identify specific topics of interest. Even if high ratings for all defined topics reflect the true preferences, individual information need should guide health care professionals in delivering the most important or urgent information in the limited consultation time [31]. Therefore, studies assessing information needs should include a prioritization of the topics by the participants. Prioritization can be achieved in several ways. One opportunity is to let them select a limited number of relevant topics out of a list of predefined topics [22]. Another way to avoid ceiling effects is the use of differential scaling to rate relative levels of importance [30]. Choi et al. have tested the consistency of breast cancer patients in prioritizing information needs making paired comparison judgments, another method to assess the priority of information needs [31]. However, the number of comparisons ascends considerably with a rising number of predefined topics.

Another potential source for differences in the results was observed between the usage of scales and other methods. The kind of questions differed in most cases. In scaling tasks participants were often asked to rate how important [13, 14, 30], interesting [18] or relevant [24] different topics are in general. Using other methods (e.g. open-ended questions, dichotomous questions) participants usually expressed their personal need for further information regarding specific topics [12, 16, 17, 21, 26]. Perhaps people rate topics as important, interesting or relevant but do not need further information because they are already well-informed or satisfied with information provision.

### Topics and extent of information needs, and associated factors

Most frequently described information needs were related to predefined topics such as treatment, diagnosis, prevention and health promotion, etiology and prognosis. However, the same topics were explicitly specified as items in qualitative studies [16, 17, 23, 24, 26].

Information needs were found in all kinds of surveyed patients as well as in samples of the general population. They were found for all the above mentioned more general topics. Regarding more specific topics such as CAM and nutrition, they were found to be of high interest in every survey containing these predefined topics. However, information about the medical system, financial and legal issues, rehabilitation and impact on social life was desired less frequently.

Several variables were found to be associated with the extent and type of information needs. A younger age, shorter duration of disease, poorer health status or higher degree of severity of a disease, higher anxiety and depression scores appear to be associated with higher unmet information needs in at least two studies, respectively. The results for the association with gender, education level and knowledge were less consistent. Knowledge or the amount of prior information does not seem to be a reliable explanatory variable for the need/desire for further information. Two studies reported that patients with higher knowledge had fewer information needs [10, 23], while other studies detected no association between knowledge and information needs [22, 24]. Emotional traits and conditions like trust, attention of nurses or the empathy of the health care professional had a strong association with the need for information [9, 17, 27]. There is some evidence of a change in the type and extent of information needs over the course of disease [26, 32].

### Comparison with other reviews

Recent reviews were restricted to certain diseases, mainly cancer, and to patients and relatives [4–6, 33]. According to our review, they found high information needs, in particular for general topics as treatment. Rutten et al. and Puts et al. also analysed the association between sociodemographic factors and information needs, and reported higher information needs in younger people in the included studies, in line with our finding [5, 6].

### Limitations

Our review has some limitations. We have no information about the specificity and sensitivity of our geographical search filter. In addition, the definition of information needs is ambiguous in relation to the terminology used. Thus, we cannot preclude having missed relevant studies. Furthermore, we did not use a definition for “health”. The choice of our databases might have resulted in a stronger focus on medical and psychological dimensions, while neglecting social dimensions of health. However, we did not experience any problems in deciding whether a study dealt with health information needs or not.

The extension of our search strategy to other databases might have yielded additional studies for inclusion. For example, CINAHL is commonly used by qualitative researchers. We also did not search for grey literature.

We only searched studies until August 2012. Further relevant studies meeting our inclusion criteria might have been published after this search date. However, following the idea of qualitative research, we feel that we reached a point of data saturation with respect to the methodological aspects of studies investigating information needs and further studies are unlikely to change our conclusion unless the methodological approaches change.

The evidence synthesis and the interpretation of our findings is hampered by the inclusion of very heterogeneous studies in terms of their objectives, methods and the investigated diseases.

There is some evidence that patients’ participation preferences, their satisfaction with health care or care provision, patients’ involvement in care decisions and their need for information differ between countries and health care systems [28, 34]. As we included studies performed in Germany, the transferability of our findings to other countries may be limited. We compared our findings to those of the few existing reviews regarding IN. However, we are not aware of any other reviews or relevant studies for direct comparison with our nation-focused results.

We did not assess quality of the included studies due to a lack of an established and validated critical appraising tool for the included study designs. However, we have considered methodological issues in the discussion section.

## Conclusion

The consideration of individual information needs is needed to provide tailored information. This is e.g. a crucial precondition of shared decision making.

Empirical findings regarding patients’ information needs are lacking for the most widespread diseases in Germany. However, our study found a considerable extent of information needs in all assessed target groups. Apart from common pre-defined topics of information needs, e.g., treatment and diagnosis, etiology, researchers, care providers and developers of patient information and decision aids should consider a wide range of potential individual topics when assessing information needs or providing information to patients, relatives, and the general population. In addition, individual characteristics should be considered when providing information. We found no study investigating the preferred formats for health-related information.

Concerning methodology, studies should not rely exclusively on ratings of predefined topics but should also ask open ended questions, seek for prioritization and apply techniques appropriate to discriminate between people’s needs for the most relevant information and different disease specific topics.

## Additional file

Additional file 1:
**Search strategy.** (DOCX 24 kb)

## Abbreviations

CAM: Complementary and alternative medicine
PP: Primary-progressive
