# Supplementary material for: Studies analysing the need for health-related information in Germany - a systematic review
Source: BMC Health Serv Res. 2015 Sep 23;15:407. doi: 10.1186/s12913-015-1076-9 (PMC4579794; doi:10.1186/s12913-015-1076-9)
Supplement: Additional file 1: — Search strategy. (DOCX 24 kb) [file 12913_2015_1076_MOESM1_ESM.docx]

**Appendix 1**

Embase and Medline (via Embase)

'information need?':ab,ti OR 'information seeking'/exp OR 'information seeking':ab,ti OR 'consumer health information'/exp OR 'consumer health information':ab,ti OR ('information'/exp OR information:ab,ti AND demand:ab,ti) OR ('information'/exp OR information:ab,ti AND requirement?:ab,ti) OR ('information'/exp OR information:ab,ti AND 'preferenc?':ab,ti) AND ('questionnaire'/exp OR questionnaire:ab,ti OR 'interview'/exp OR interview:ab,ti OR 'survey health'/exp OR 'survey health':ab,ti OR survey OR survey:ab,ti) AND ('germany'/exp OR germany:ca,ad,ab,ti OR german:ca,ad,ab,ti OR gkv:ab,ti OR deutschland:ca,ad,ab,ti OR deutsch:ca,ad,ab,ti OR berlin:ca,ad,ab,ti OR hamburg:ca,ad,ab,ti OR münchen:ca,ad,ab,ti OR muenchen:ca,ad,ab,ti OR munich:ca,ad,ab,ti OR köln:ca,ad,ab,ti OR koeln:ca,ad,ab,ti OR cologne:ca,ad,ab,ti OR frankfurt:ca,ad,ab,ti OR stuttgart:ca,ad,ab,ti OR düsseldorf:ca,ad,ab,ti OR duesseldorf:ca,ad,ab,ti OR dortmund:ca,ad,ab,ti OR essen:ca,ad,ab,ti OR bremen:ca,ad,ab,ti OR dresden:ca,ad,ab,ti OR leipzig:ca,ad,ab,ti OR hannover:ca,ad,ab,ti OR nürnberg:ca,ad,ab,ti OR Aachen:ca,ad,ab,ti OR augsburg:ca,ad,ab,ti OR bamberg:ca,ad,ab,ti OR bayreuth:ca,ad,ab,ti OR benediktbeuern:ca,ad,ab,ti OR bochum:ca,ad,ab,ti OR braunschweig:ca,ad,ab,ti OR chemnitz:ca,ad,ab,ti OR clausthal:ca,ad,ab,ti OR cottbus:ca,ad,ab,ti OR darmstadt:ca,ad,ab,ti OR detmold:ca,ad,ab,ti OR Eichstätt:ca,ad,ab,ti OR Eichstaett:ca,ad,ab,ti OR Ingolstadt:ca,ad,ab,ti OR Erfurt:ca,ad,ab,ti OR Erlangen:ca,ad,ab,ti OR flensburg:ca,ad,ab,ti OR freiberg:ca,ad,ab,ti OR freiburg:ca,ad,ab,ti OR friedrichshafen:ca,ad,ab,ti OR fulda:ca,ad,ab,ti OR Gießen:ca,ad,ab,ti OR Giessen:ca,ad,ab,ti OR Greifswald:ca,ad,ab,ti OR Göttingen:ca,ad,ab,ti OR Goettingen:ca,ad,ab,ti OR Hagen:ca,ad,ab,ti OR Halle:ca,ad,ab,ti OR heidelberg:ca,ad,ab,ti OR Hildesheim:ca,ad,ab,ti OR Ilmenau:ca,ad,ab,ti OR Jena:ca,ad,ab,ti OR Kaiserslautern:ca,ad,ab,ti OR Karlsruhe:ca,ad,ab,ti OR Kassel:ca,ad,ab,ti OR Kiel:ca,ad,ab,ti OR Koblenz:ca,ad,ab,ti OR Konstanz:ca,ad,ab,ti OR Luebeck:ca,ad,ab,ti OR Ludwigsburg:ca,ad,ab,ti OR Lübeck:ca,ad,ab,ti OR Lueneburg:ca,ad,ab,ti OR Lüneburg:ca,ad,ab,ti OR Mainz:ca,ad,ab,ti OR Marburg:ca,ad,ab,ti OR Münster:ca,ad,ab,ti OR muenster:ca,ad,ab,ti OR neuendettelsau:ca,ad,ab,ti OR oestrich-winkel:ca,ad,ab,ti OR oldenburg:ca,ad,ab,ti OR osnabrueck:ca,ad,ab,ti OR osnabrück:ca,ad,ab,ti OR paderborn:ca,ad,ab,ti OR passau:ca,ad,ab,ti OR potsdam:ca,ad,ab,ti OR regensburg:ca,ad,ab,ti OR rostock:ca,ad,ab,ti OR saar:ca,ad,ab,ti OR Augustin:ca,ad,ab,ti OR Schwäbisch:ca,ad,ab,ti OR Gmünd:ca,ad,ab,ti OR schwaebisch:ca,ad,ab,ti OR gmuend:ca,ad,ab,ti OR siegen:ca,ad,ab,ti OR speyer:ca,ad,ab,ti OR trier:ca,ad,ab,ti OR trossingen:ca,ad,ab,ti OR tuebingen:ca,ad,ab,ti OR Tübingen:ca,ad,ab,ti OR ulm:ca,ad,ab,ti OR vallendar:ca,ad,ab,ti OR vechta:ca,ad,ab,ti OR weimar:ca,ad,ab,ti OR weingarten:ca,ad,ab,ti OR Witten:ca,ad,ab,ti OR wuppertal:ca,ad,ab,ti OR wuerzburg:ca,ad,ab,ti OR Würzburg:ca,ad,ab,ti OR zittau:ca,ad,ab,ti OR nuernberg:ca,ad,ab,ti OR duisburg:ca,ad,ab,ti OR bochum:ca,ad,ab,ti OR wuppertal:ca,ad,ab,ti OR bonn:ca,ad,ab,ti OR bielefeld:ca,ad,ab,ti OR mannheim:ca,ad,ab,ti OR 'north rhine-westphalia':ca,ad,ab,ti OR nrw:ca,ad,ab,ti OR 'nordrhein westfalen':ca,ad,ab,ti OR 'rhine ruhr':ca,ad,ab,ti OR rhein:ca,ad,ab,ti OR ruhr:ca,ad,ab,ti OR 'schleswig holstein':ca,ad,ab,ti OR 'mecklenburg vorpommern':ca,ad,ab,ti OR 'mecklenburg-western pomerania':ca,ad,ab,ti OR brandenburg:ca,ad,ab,ti OR sachsen:ca,ad,ab,ti OR saxony:ca,ad,ab,ti OR 'saxony anhalt':ca,ad,ab,ti OR 'sachsen anhalt':ca,ad,ab,ti OR thuringia:ca,ad,ab,ti OR thüringen:ca,ad,ab,ti OR thueringen:ca,ad,ab,ti OR niedersachsen:ca,ad,ab,ti OR 'lower saxony':ca,ad,ab,ti OR hesse:ca,ad,ab,ti OR hessia:ca,ad,ab,ti OR hessen:ca,ad,ab,ti OR 'rhineland palatinate':ca,ad,ab,ti OR rheinland:ca,ad,ab,ti OR pfalz:ca,ad,ab,ti OR saarland:ca,ad,ab,ti OR baden:ca,ad,ab,ti OR württemberg:ca,ad,ab,ti OR wuerttemberg:ca,ad,ab,ti OR bavaria:ca,ad,ab,ti OR bayern:ca,ad,ab,ti) AND [2000-2013]/py

**Psycinfo (via EBSCO)**

((AB information need? OR TI information need?) OR (SU information seeking OR TI information seeking OR AB information seeking) OR (SU consumer health information OR TI consumer health information OR AB consumer health information) OR ((SU information OR TI information OR AB information) AND (AB demand OR TI demand)) OR ((SU information OR TI information OR AB information) AND (AB requirement? OR TI requirement?)) OR ((SU information OR TI information OR AB information) AND (AB preferenc? OR TI preferenc?)) AND

((SU questionnaire OR AB questionnaire OR TI questionnaire) OR (SU interview OR TI interview OR AB interview) OR (SU survey health OR TI survey health OR AB survey health) OR (SU survey OR AB survey OR TI survey))

AND ((SU germany OR AB germany OR TI germany) OR (AB german OR TI german) OR (AB GKV OR TI GKV) OR (AB deutschland OR TI deutschland) OR (AB deutsch OR TI deutsch) OR (TI berlin OR AB Berlin) OR (TI hamburg OR AB hamburg) OR (AB münchen OR TI münchen) OR (AB muenchen OR TI muenchen) OR (AB munich OR TI munich) OR (AB köln OR TI köln) OR (TI koeln OR AB koeln) OR (AB cologne OR TI cologne) OR (AB frankfurt OR TI frankfurt) OR (AB stuttgart OR TI stuttgart) OR (AB düsseldorf OR TI düsseldorf) OR (AB duesseldorf OR TI duesseldorf) OR (AB dortmund OR TI dortmund) OR (AB essen OR TI essen) OR (AB bremen OR TI bremen) OR (AB dresden OR TI dresden) OR (AB leipzig OR TI leipzig) OR (AB hannover OR TI hannover) OR (AB nürnberg OR TI nürnberg) OR (AB nuernberg OR TI nuernberg) OR (AB Aachen OR TI Aachen) OR (AB augsburg OR TI Ausgburg) OR (AB bamberg OR TI bamberg) OR (AB bayreuth OR TI bayreuth) OR (AB benediktbeuern OR TI benediktbeuern) OR (AB bochum OR TI bochum) OR (AB braunschweig OR TI braunschweig) OR (AB chemnitz OR TI chemnitz) OR (AB clausthal OR TI clausthal) OR (TI cottbus OR AB cottbus) OR (AB darmstadt OR TI darmstadt) OR (AB detmold OR TI detmold) OR (TI Eichstätt OR AB Eichstätt) OR (TI Eichstaett OR AB Eichstaett) OR (AB Ingolstadt OR TI Ingolstadt) OR (AB Erfurt OR TI Erfurt) OR (AB Erlangen OR TI Erlangen) OR (TI flensburg OR AB flensburg) OR (AB freiberg OR TI freiberg) OR (AB freiburg OR TI freiburg) OR (AB friedrichshafen OR TI friedrichshafen) OR (AB fulda OR TI fulda) OR (TI Gießen OR AB Gießen) OR (AB Giessen OR TI Giessen) OR (AB Greifswald OR TI Greifswald) OR (AB Göttingen OR TI Göttingen) OR (AB Goettingen OR TI Goettingen) OR (AB Hagen OR TI Hagen) OR (AB Halle OR TI Halle) OR (AB heidelberg OR TI heidelberg) OR (AB Hildesheim OR TI hildesheim) OR (AB Ilmenau OR TI ilmenau) OR (TI Jena OR AB Jena) OR (AB Kaiserslautern OR TI kaiserslautern) OR (AB Karlsruhe OR TI karlsruhe) OR (AB Kassel OR TI kassel) OR (AB Kiel OR TI kiel) OR (AB Koblenz OR TI koblenz) OR (AB Konstanz OR TI konstanz) OR (AB Luebeck OR TI luebeck) OR (TI Ludwigsburg OR AB Ludwigsburg) OR (AB Lübeck OR TI Lübeck) OR (AB Lueneburg OR TI Lueneburg) OR (AB Lüneburg OR TI Lüneburg) OR (TI Mainz OR AB mainz) OR (TI Marburg OR AB Marburg) OR (TI Münster OR AB muenster) OR (TI muenster OR AB muenster) OR (TI neuendettelsau OR AB neuendettelsau) OR (TI oestrich-winkel OR AB oestrich-winkel) OR (TI oldenburg OR AB oldenburg) OR (AB osnabrueck OR TI osnabrueck) OR (TI osnabrück OR AB osnabrück) OR (AB paderborn OR TI paderborn) OR (TI passau OR AB passau) OR (AB potsdam OR TI potsdam) OR (AB regensburg OR TI regensburg) OR (AB rostock OR TI rostock) OR (AB saar OR TI saat) OR (TI Augustin OR AB Augustin) OR (TI Schwäbisch Gmünd OR AB Schwäbisch Gmünd) OR (TI Schwaebisch Gmuend OR AB schwaebisch gmuend) OR (AB siegen OR TI siegen) OR (AB speyer OR TI speyer) OR (AB trier OR TI trier) OR (TI trossingen OR AB trossingen) OR (TI tuebingen OR AB tuebingen) OR (TI Tübingen OR AB Tübingen) OR (TI ulm OR AB ulm) OR (TI vallendar OR AB vallendar) OR (AB vechta OR TI vechta) OR (AB weimar OR TI weimar) OR (AB weingarten OR TI weingarten) OR (TI Witten OR AB Witten) OR (TI wuppertal OR AB wuppertal) OR (AB wuerzburg OR TI wuerzburg) OR (TI Würzburg OR AB Würzburg) OR (TI zittau OR AB zittau) OR (AB duisburg OR TI duisburg) OR (AB bochum OR TI bochum) OR (AB wuppertal OR TI wuppertal) OR (AB bonn OR TI bonn) OR (TI bielefeld OR AB bielefeld) OR (AB mannheim OR TI mannheim) OR (TI north rhine-westphalia OR AB north rhine-westphalia) OR (AB nrw OR TI nrw) OR (AB nordrhein westfalen OR TI nordrhein westfalen) OR (AB rhine ruhr OR TI rhine ruhr) OR (TI rhein OR AB rhein) OR (TI ruhr OR AB ruhr) OR (AB schleswig holstein OR TI schleswig holstein) OR (AB mecklenburg vorpommern OR TI mecklenburg vorpommern) OR (TI mecklenburg-western pomerania OR AB mecklenburg-western pomerania) OR (AB brandenburg OR TI brandenburg) OR (AB sachsen OR TI sachsen) OR (AB saxony OR TI saxony) OR (TI saxony anhalt OR AB saxony anhalt) OR (AB sachsen anhalt OR TI sachsen anhalt) OR (AB thuringia OR TI thuringia) OR (AB thüringen OR TI thüringen) OR (AB thueringen OR TI thueringen) OR (TI niedersachsen OR AB niedersachsen) OR (AB lower saxony OR TI lower saxony) OR (AB hesse OR TI hesse) OR (AB hessia OR TI hessia) OR (TI hessen OR AB hessen) OR (AB rhineland palatinate OR TI rhineland palatinate) OR (AB rheinland OR TI rheinland) OR (AB pfalz OR TI pfalz) OR (AB saarland OR TI saarland) OR (AB baden OR TI baden) OR (AB württemberg OR TI württemberg) OR (AB wuerttemberg OR TI wuerttemberg) OR (AB bavaria OR TI bavaria) OR (AB bayern OR TI bayern))

**Cochrane Library**

((“information need?”:ab,ti) OR (“information seeking behavior”/exp OR “information seeking”:ti,ab) OR (“consumer health information”/exp) OR (information:ab,ti AND demand:ab,ti) OR (information:ab,ti AND requirement?:ab,ti) OR (information:ab,ti AND “preferenc?”:ab,ti)) AND ((„questionnaires“/exp OR questionnaire?:ab,ti) OR („MeSH descriptor Interviews as Topic“/exp OR interview?:ab,ti) OR („health surveys“/exp) OR (survey OR survey:ab,ti)) AND ((„germany“/exp OR germany:ab,ti) OR german:ab,ti OR GKV:ab,ti OR deutschland:ab,ti OR deutsch:ti,ab OR berlin:ab,ti OR hamburg:ab,ti OR münchen:ab,ti OR muenchen:ab,ti OR munich:ab,ti OR köln:ab,ti OR koeln:ab,ti OR cologne:ab,ti OR frankfurt:ab,ti OR stuttgart:ab,ti OR düsseldorf:ab,ti OR duesseldorf:ab,ti OR dortmund:ab,ti OR essen:ab,ti OR bremen:ab,ti OR dresden:ab,ti OR leipzig:ab,ti OR hannover:ab,ti OR nürnberg:ab,ti OR nuernberg:ab,ti OR duisburg:ab,ti OR bochum:ab,ti OR wuppertal:ab,ti OR bonn:ab,ti OR bielefeld:ab,ti OR mannheim:ab,ti OR „north rhine-westphalia“:ab,ti OR nrw:ab,ti OR „nordrhein westfalen“:ab,ti OR „rhine ruhr“:ab,ti OR rhein:ab,ti OR ruhr:ab,ti OR „schleswig holstein“:ab,ti OR „mecklenburg vorpommern“:ab,ti OR „mecklenburg-western pomerania“:ab,ti OR brandenburg:ab,ti OR sachsen:ab,ti OR saxony:ab,ti OR „saxony anhalt“:ab,ti OR „sachsen anhalt“:ab,ti OR thuringia:ab,ti OR thüringen:ab,ti OR thueringen:ab,ti OR niedersachsen:ab,ti OR „lower saxony“:ab,ti OR hesse:ab,ti OR hessia:ab,ti OR hessen:ab,ti OR „rhineland palatinate“:ab,ti OR rheinland:ab,ti OR pfalz:ab,ti OR saarland:ab,ti OR baden:ab,ti OR württemberg:ab,ti OR wuerttemberg:ab,ti OR bavaria:ab,ti OR bayern:ab,ti))
